# Can herpes simplex virus type 2 suppression slow HIV disease progression: a study protocol for the VALacyclovir In Delaying Antiretroviral Treatment Entry (VALIDATE) trial

**DOI:** 10.1186/1745-6215-11-113

**Published:** 2010-11-24

**Authors:** Darrell HS Tan, Janet M Raboud, Rupert Kaul, Beatriz Grinsztejn, Pedro Cahn, Sharon L Walmsley

**Affiliations:** 1 University Health Network, 585 University Ave., 13N - 1323, Toronto, Ontario, M5G 2N2 Canada; 2 Instituto de Pesquisa Clínica Evandro Chagas (IPEC), Fundação Oswaldo Cruz, Avenida Brasil, 4365 - Manguinhos, 21040-900, Rio de Janeiro - RJ - Brazil; 3 Fundación Huesped, Angel Peluffo 3932, Buenos Aires, Argentina, C1202ABB

## Abstract

**Background:**

Although highly active antiretroviral therapy (HAART) has dramatically decreased HIV-related morbidity and mortality, the associated costs, toxicities, and resistance risks make the potential delay of HAART initiation an attractive goal. Suppression of herpes simplex virus type 2 (HSV-2) may be a novel strategy for achieving this goal because HSV-2 is associated with clinically significant increases in HIV viral load, the primary driver of HIV disease progression.

**Methods/Design:**

The VALacyclovir In Delaying Antiretroviral Treatment Entry (VALIDATE) trial is a multicentre, randomized, fully blinded, clinical trial of twice daily valacyclovir 500 mg versus placebo for delaying the need for initiating HAART among HIV-1, HSV-2 co-infected HAART-naïve adults. 480 participants from Canada, Brazil and Argentina will undergo quarterly clinical follow-up until reaching the composite primary endpoint of having a CD4+ T-cell count ≤ 350 cells/mm^3 ^or initiation of HAART for any reason, whichever occurs first. The primary analysis will use a proportional hazards model, stratified by site, to estimate the relative risk of progression to this endpoint associated with valacyclovir. Secondary analyses will compare the rates of change in CD4 count, median log_10 _HIV viral load, drug-related adverse events, frequency of HSV reactivations, rate of acyclovir-resistant HSV, and quality of life between study arms.

**Discussion:**

Although HIV treatment guidelines continue to evolve, with some authorities recommending earlier HAART among asymptomatic individuals, the potential delay of HAART remains a clinically relevant goal for many. If shown to be of benefit, implementation of the VALIDATE intervention will require careful consideration of both individual patient-level and public health implications.

**Trial Registration:**

Current Controlled Trials ISRCTN66756285

ClinicalTrials.gov NCT00860977

## Background

Highly active antiretroviral therapy (HAART) has dramatically reduced morbidity and mortality related to HIV-1 infection (herein referred to as 'HIV'), transforming an invariably fatal disease into a manageable, chronic condition. Although debate continues as to the advantages and disadvantages of earlier HAART initiation, the cost, potential long and short-term toxicities, and risk of developing drug-resistant HIV associated with daily, lifelong HAART would make the delay of HAART initiation an attractive goal for many patients. Suppression of herpes simplex virus (HSV)-2 co-infection may provide a novel therapeutic strategy for achieving this goal. The VALacyclovir In Delaying Antiretroviral Treatment Entry (VALIDATE) trial has been designed to address this question.

Recommendations for initiating HAART are based on the natural history of HIV infection and clinical trials of HIV therapy. After acute infection there is an initial profound drop in the CD4-positive T lymphocyte count from levels of > 1000 cells/mm^3 ^to roughly 700 cells/mm^3 ^six months later[1]. Patients then enter a generally asymptomatic period characterized by a steady CD4 count decline of about 50-60 cells/mm^3 ^per year, although this varies according to the HIV RNA viral load set point[2-5]. The risk of opportunistic infection and mortality rises sharply at CD4 counts ≤ 200 cells/mm^3^. Current guidelines therefore agree that HAART is warranted when the CD4 count falls to 350 cells/mm^3 ^in asymptomatic, non-pregnant adults; debate continues on the role of earlier treatment initiation, with some but not all guidelines recommending treatment at a threshold of 500 cells/mm^3 ^[6-9]. Once HAART is initiated, treatment must be lifelong, because HIV cannot be eradicated, and because treatment interruptions are associated with increased rates of HIV-associated and non-HIV-associated conditions[10].

HSV-2 is among the most common co-infections in HIV, with a prevalence of 52-95%,[11-16] and several important interactions have been observed between these two viruses. First, HSV-2 is associated with increased HIV acquisition and transmission, even in asymptomatic individuals[17-27]. Recent trials of daily acyclovir for HSV-2 suppression, however, did not show a benefit on decreasing HIV acquisition or transmission[28-30]. Persistence of HIV target cells in the genital tract, suboptimal dosing and inadequate adherence may have contributed to the negative results[31]. Second, both symptomatic, clinical recurrences of HSV-2, and asymptomatic shedding of HSV-2 have been clearly associated with increased HIV levels in both genital secretions and plasma[32-35]. Upregulation of HIV replication by HSV gene products such as infected cell protein (ICP)-0, ICP-4, ICP-27 and Us11, and stimulation of the NF-Kappa B pathway may contribute to these increases in HIV viral load[36-40]. Further, clinical trials using both acyclovir and valacyclovir suppressive therapy in co-infected individuals, including 20 men who have sex with men (MSM) in Peru, 140 women in Burkina Faso, and 67 women in Thailand have demonstrated clinically significant, reciprocal decreases in HIV RNA viral load of 0.33-0.53 log_10_[41-43].

The plasma HIV viral load, in turn, is the major predictor of CD4 cell decline and HIV disease progression, and viral load strata that increase by about 0.5 log copies/mL correlate with increased rates of CD4 decline of roughly 10 cells/mm^3^/year[2-5]. It follows, then, that HSV-2 suppressive therapy might delay HIV disease progression in HAART-untreated individuals through its effects on the HIV viral load. Indeed, a meta-analysis from the pre-HAART era suggested that > 3200 mg per day of acyclovir offered a significant survival benefit (incidence rate ratio for mortality, 0.81, 95%CI 0.68-0.96), although this effect was largely related to treatment of active herpesvirus disease in individuals with late-stage HIV/AIDS[44]. More recently, the multicentre Partners in Prevention trial from Sub-Saharan Africa has shown that acyclovir 400 mg twice daily was associated with moderately decreased progression to the composite endpoint of CD4 < 200 cells/mm3, HAART initiation, or non-trauma related death (hazard ratio = 0.84, 95%CI 0.71-0.98)[45].

These results bolster interest in the VALIDATE trial, which is addressing related but complementary issues. First, given the conduct of the Partners trial in resource-limited settings, it remains important to study the impact of HSV-2 suppression in co-infected patients from industrialized and middle-income countries. The endpoint of the Partners trial represented a stage of disease that can be avoided in settings where the standard of care is to offer HAART earlier; it is encouraging, therefore, that an exploratory analysis from the Partners trial demonstrated a delay in achieving CD4 < 350 cells/mm^3 ^from acyclovir as well (HR = 0.81, 95%CI 0.71-0.93, p = 0.002). Further, rates of CD4 count decline may be different in Sub-Saharan Africa due to the effects of nutritional status, heightened levels of immune activation (for example, as a result of co- or intercurrent infections), and HIV clade[46-49]. The predominant clades in Sub-Saharan Africa (A, D and C) differ from those in most industrialized countries (B and some others) and in South America (B but some F, C and recombinants)[50,51]. In addition, it should be noted that disease progression was a secondary endpoint of the Partners trial, which was primarily designed to study the impact of HSV-2 suppression on HIV transmission. Finally, VALIDATE is employing valacyclovir instead of acyclovir for HSV-2 suppression, and the dose of 500 mg twice daily has been demonstrated to achieve excellent herpes suppression in clinical trials of HIV, HSV-2 co-infected adults[41,42]. Valacyclovir is the pro-drug of acyclovir, and may offer advantages over acyclovir owing to an improved bioavailability (54.5% versus 15-30%) and lower pill burden[52].

Chronic suppressive therapy with valacyclovir is safe, well-tolerated and already in widespread use to prevent HSV outbreaks in those with frequent (five times a year or more) clinical reactivation. However, up to 90% of individuals with HSV-2 infection do not have any symptoms at all[53]. If HIV co-infected, such individuals may benefit from HSV-2 suppressive therapy not only through its effects on reducing herpes transmission and herpes reactivation, [54-56] but also through its salutary effects on HIV. The purpose of this multicentre, randomized, placebo-controlled clinical trial is therefore to answer the question, "Among adults co-infected with HIV and HSV-2 who are not using chronic suppressive anti-HSV therapy and who are neither currently using nor deemed to require HAART, can oral valacyclovir 500 mg twice daily delay the time until the need for HAART initiation?"

## Methods

### Study Design

The VALIDATE study is a multicentre, randomized, placebo-controlled, fully blinded, clinical trial of twice daily oral valacyclovir 500 mg versus placebo with the goal of delaying the need for initiating HAART among HIV, HSV-2 co-infected individuals who neither use nor require HAART, and who have not used chronic suppressive anti-HSV therapy for at least the 6 months prior to study initiation.

### Study Objectives

The primary objective of this trial is to compare the time from enrolment to the primary endpoint of either a CD4 cell count ≤ 350 cells/mm^3 ^measured on two consecutive occasions at least 1 month apart, or initiation of HAART for any reason, between adults with both stable untreated HIV and HSV-2 co-infection who are randomized to valacyclovir compared to those randomized to placebo.

Secondary objectives are as follows:

• To compare the annual rate of change in the absolute CD4 cell count between study arms.

• To compare the annual rate of change in the CD4 cell count percentage between study arms.

• To compare the median log_10 _HIV viral load between study arms.

• To compare the rate of drug-related adverse events between study arms.

• To compare the median frequency of HSV reactivations per year between study arms.

• To compare the rate of acyclovir-resistant HSV between study arms.

• To compare quality of life between study arms.

A substudy will characterize plasma markers of inflammation in this cohort of HAART-naive HIV-infected patients and compare the levels of these markers between study arms.

### Study Hypotheses

The primary hypothesis of the trial is that valacyclovir 500 mg orally twice daily will delay the time until HAART is initiated among adults co-infected by stable untreated HIV and HSV type 2 co-infection. The secondary hypotheses are that valacyclovir 500 mg orally twice daily is associated with a slower rate of change in both the absolute CD4 cell count and the CD4 cell count percentage, a lower median log_10 _HIV viral load, a similar rate of drug-related adverse events, a lower frequency of HSV reactivations, a similar rate of acyclovir-resistant HSV, and improvements in quality of life compared with placebo.

### Eligibility Criteria

Individuals will be eligible for the trial if they meet the following inclusion criteria:

• adult (aged 18 years or older or as per Local/Provincial Guidelines)

• documented HIV-1 infection (determined by EIA and Western blot)

• documented HSV-2 seropositivity (determined by ELISA during screening; see below)

• no use of chronic anti-HSV therapy for the past 6 months, and not anticipated to require chronic anti-HSV therapy during the study

• antiretroviral naïve (no more than 14 days of total prior ARV exposure)

• CD4 count within the 400-900 cells/mm^3 ^range (inclusive) on two consecutive occasions, with at least one measurement within 4 weeks of initiating trial

• does not meet recommendations for initiating ARV therapy according to current guidelines[57,58]

Chronic anti-HSV therapy refers to the regular, daily use of medications with anti-HSV activity (such as acyclovir, valacyclovir, famciclovir, ganciclovir, valganciclovir, cidofovir, foscarnet). It does not refer to the episodic use of short courses of these medications for treatment of isolated HSV flares for a few days at a time.

The exclusion criteria for this study are as follows:

• pregnancy or actively planning to become pregnant

• receiving chemotherapy, chronic steroid therapy or other immunomodulatory medications (e.g. interferon, azathioprine, methotrexate, TNF-alpha antagonists, etc.)

• Estimated creatinine clearance < 30 mL/min

• Other medical condition likely to cause death within 24 months

• Enrolled in a therapeutic vaccine or immunotherapy trial

• Enrolled in another trial investigating the impact of another intervention on HIV disease progression

• HIV elite controller (EC), phenotypically defined here as documented duration of HIV infection of ≥5 years, a persistent CD4 cell count ≥500 cells/mm^3^, and a persistent plasma HIV viral load of < 1000 copies/mL in the absence of antiretroviral therapy[3,59-61]

A stringent, empiric definition of the EC phenotype will be used because previous reports have primarily characterized ECs on the basis of genetic and immune markers beyond the means of this study[3,59-61]. The heterogeneity of CD4 counts and viral loads in ECs implies that a more permissive definition may overlap considerably with the general HIV-infected population and risk introducing a selection bias.

### Study setting

The VALIDATE trial will be conducted in industrialized/middle-income country settings. The study will randomize 480 participants, 240 in each arm. Participants will be recruited from sites including clinical research sites of the CIHR Canadian HIV Trials Network (CTN), the Instituto de Pesquisa Clinica Evandro Chagas (IPEC) HIV/AIDS Clinical Research Center of the Oswaldo Cruz Foundation in Rio de Janeiro, Brazil, and the Hospital Fernandez/Fundación Huesped in Buenos Aires, Argentina. The CTN is a nationwide partnership established in 1990 of over 30 clinical trial sites across Canada, including both university-affiliated and community-based HIV clinics, with extensive experience in conducting HIV clinical trials. Both the IPEC Centre in Rio and the Hospital Fernandez and affiliated Fundación Huesped in Buenos Aires are national reference centres for infectious disease research, care and training, and are experienced leaders in international HIV/AIDS clinical trials.

### Treatment Groups

Participants in the intervention group will receive oral valacyclovir 500 mg twice daily, the standard dose used for HSV-2 suppression in HIV-infected individuals[15,62]. Valacyclovir is safe and well tolerated, as evidenced by clinical trials, where side effects have been reported at rates no different from those of placebo, and clinical experience[54,55,63,64]. Individuals in the control arm will receive an odourless placebo tablet identical to valacyclovir in appearance and taste, to be taken twice daily. Use of other anti-HSV medication (including topical acyclovir) during the trial is prohibited, except during treatment of herpes flares (described below).

### Randomization and blinding methods

Randomization is stratified by site in permuted blocks of variable size (4 and 6) using a computer-generated list of random numbers. The CTN created and coordinated the allocation sequence, and randomization is performed through a secure website which ensures allocation concealment. All participants and study personnel (including investigators, research coordinators, data analysts) will be blinded to treatment allocation throughout the trial. Access to the allocation code will be restricted to one study statistician who will not perform the final study analyses.

### Outcome Measures

The primary outcome is the time from baseline until reaching the primary endpoint, a composite of either a CD4 cell count ≤ 350 cells/mm^3 ^measured on two consecutive occasions at least 1 month apart, or initiation of HAART for any reason, whichever occurs first. This endpoint was selected because reaching the treatment threshold for initiating HAART is an important clinical event signifying the need for lifelong therapy. The CD4 count of ≤ 350 cells/mm^3 ^must be confirmed by a second measurement to verify the CD4 count is within the treatment range before initiating therapy. The primary endpoint also includes the initiation of HAART for any reason, since patients who start HAART outside these guidelines are also committed to lifelong therapy. This group may include individuals who choose to start therapy at earlier stages of disease (e.g. because of high viral load set-point), and some who develop HIV-related complications (e.g. tuberculosis) at higher CD4 cell counts, among others. For example, women who initiate HAART during pregnancy primarily for the prevention of mother-to-child transmission of HIV, and hepatitis B co-infected individuals who initiate HAART primarily for the purposes of treating their hepatitis B infection, will also be considered to have met the primary endpoint. It was important not to restrict the primary endpoint to initiation of HAART alone, since some patients may not initiate HAART despite reaching treatment thresholds for reasons including patient anxiety, concerns about adherence, and lack of familiarity with guidelines[65].

HAART initiation guidelines are continuously under evaluation, and HIV treatment guidelines may change. However, a change will not threaten the integrity of the study for a number of reasons. First, guidelines on HAART initiation have recently been revised[6,9]. Recommendations to uniformly increase the treatment threshold above 350 cells/mm^3 ^in both industrialized and middle-income settings based on definitive clinical trial data are not anticipated during the course of the trial. Second, as described above, the primary endpoint of this study is a composite of not only meeting the current CD4 threshold of 350 cells/mm^3^, but also the initiation of HAART for any reason, whichever occurs first. Finally, randomization in this trial will be stratified by site to help account for variation in practice regarding initiation of HAART across sites.

The secondary outcome measures to be compared between the two treatment groups include:

• Annual rate of change in CD4 count, calculated as the slope of participants' CD4 count change/time

• Annual rate of change in the CD4 cell count percentage, calculated as the slope of the participants' CD4 count percentage change over time

• Log_10 _plasma HIV viral load at 12, 24 and 36 months of follow-up

• Treatment-emergent adverse events and laboratory abnormalities (CBC, plasma creatinine)

• Frequency of episodes of symptomatic HSV reactivation at any anatomic site

• Proportion of microbiologically confirmed flares of HSV during the trial that are caused by laboratory-confirmed acyclovir-resistant HSV

• Overall quality of life as measured by the MOS-HIV questionnaire at each 6-monthly time point.

### Duration of Study Participation

Study visits will occur every 3 months over 2 years of participant accrual and 3 years of follow-up. Once enrolled, participants will enter the follow-up period during which they will be treated with either valacyclovir or placebo, and undergo follow-up visits every 3 months. Follow-up for an individual participant will continue until the end of the 5-year study period unless the primary endpoint is reached first. This means that all participants will be followed until the 5-year trial ends, unless they reach a primary endpoint first.

### Safety

Adverse events and serious adverse events will be assessed at each study visit, graded on a 4-point scale (Division of AIDS Table for Grading the Severity of Adult and Pediatric Adverse Events), and assessed for their relationship to the study medication/intervention. The Data Safety Monitoring Committee (DSMC) of the CIHR CTN will be responsible for safety monitoring in this trial, and will also conduct safety analyses based on serious adverse events every 6 months, and ad hoc when necessary. The DSMC consists of clinicians, biostatisticians and a community member, from Canada and the United States.

### Adherence Assessments

Adherence will be assessed at each visit through pill counts and through a self-report questionnaire (modified from the ACTG Adherence Questionnaire) at each visit[66,67]. In addition, urine samples will be collected from all study participants at pre-planned visits, stored at -80°C, and tested for acyclovir by mass spectrometry[68] to obtain overall estimates of adherence or cross over. Testing will be performed at the conclusion of the trial to help maintain blinding.

### Management of symptomatic herpes during the trial

If a clinical reactivation of herpes symptoms occurs during the trial, participants are instructed to continue their study drug, present for clinical assessment, have samples taken to attempt microbiologic confirmation of the diagnosis where resources permit, and consider optional open-label treatment. Although participants in the treatment arm may thus receive a total valacyclovir dose of up to 3 g per day for 5 days, this quantity remains well within the safe therapeutic window of this drug. Of note, the occurrence of herpes flares in a study participant does not in itself unblind the study, because a small proportion of individuals taking chronic suppressive anti-HSV medications can experience herpes reactivations. If HSV resistance is suspected, specimens will be tested for acyclovir resistance using a plaque reduction assay[69].

### Ethical approval

Ethical approval was obtained from the Research Ethics Board of the primary study site, the University Health Network (REB# 09-0587-B), prior to commencement of study activities at any site. Each study site also obtained approval from the local research ethics board prior to initiation of the study at that site. The research is being conducted in accordance with the Declaration of Helsinki.

### Sample Size Calculation

It is hypothesized that suppression of HSV-2 using valacyclovir will increase the time until participants' CD4 counts reach ≤ 350 cells/mm^3 ^or initiate HAART from the expected value of 2 years to 2.75 years (see justification below). Participants will be recruited over 2 years and followed over 3 years. Using a conservative estimate that 25% of the sample will be lost to follow-up on average half way through the 3-year follow-up period, the expected years of follow-up is 0.75 × 3 years + 0.25 × 1.5 years = 2.625 years overall. In order to detect a difference of 0.75 years with a two-sided α = 0.0492 (adjusted for multiple analyses by the method of O'Brien and Fleming)[70] and 80% power in a survival analysis, 240 individuals will be required in each study arm, or 480 participants overall[71].

The expected rate at which HIV disease would progress in HAART-untreated individuals was based on both a retrospective review of patient charts and the published literature. Among 142 patients with at least 3 CD4 counts before starting HAART, with baseline CD4 count > 400 cells/mm^3 ^at the Toronto General Hospital Immunodeficiency Clinic, the median time from first presentation to reaching CD4 ≤ 350 cells/mm^3 ^was 2.0 years (Raboud JM, Li M: personal communication). In the subset with higher baseline CD4 counts (> 500 cells/mm^3^), the median time was 2.5 years. Consistent with these findings, a published retrospective observational cohort study of 4268 HIV-infected individuals in the United Kingdom with baseline CD4 counts > 500 cells/mm^3 ^also found the median time to until reaching CD4 counts ≤ 350 cells/mm^3 ^or initiation of antiretroviral therapy was 2.5 years[72]. Both are consistent with the generally accepted rate of CD4 count decline of 50-60 cells/mm^3 ^per year[2,3].

Valacyclovir 500 mg twice daily is estimated to decrease HIV plasma viral load by 0.5 log copies/mL. In previous studies, the magnitude of increase in HIV viral load associated with HSV-2 infection and the reciprocal decrease with valacyclovir 500 mg twice daily therapy was 0.33-0.53 log_10 _copies/mL[18,32,33,41,42]. Further, the impact on HIV viral load was more pronounced with time and in those with higher baseline CD4 counts. The median baseline CD4 counts in these trials were 226 (IQR 145-389) and 446 cells/mm^3 ^(IQR 334-628) respectively[41,42]. Among persons with higher baseline CD4 counts of 400-900 cells/mm^3^, valacyclovir is anticipated to reduce HIV viral load by about 0.5 log_10 _copies/mL. Data from large cohort studies of untreated HIV infection that correlate HIV viral load set-points with rates of CD4 cell decline [2,3] suggest that valacyclovir will attenuate the rate of CD4 count decline by 10 cells/mm^3^/year. Over the 3-5 years of follow-up in this study, the treatment arm is thus expected to experience an overall attenuation in CD4 count decline of 30-50 cells/mm^3^, roughly equivalent to the CD4 count decline seen in 9 months. The sample size calculation is based on a prolongation in the time until CD4 ≤ 350 or HAART initiation from 2 to 2.75 years. The Kaplan-Meier estimates of the proportion of ART-naïve patients who experience a fall in CD4 count to ≤ 350 cells/mm^3 ^or who start HAART in the UK study cited above are consistent with these figures[72].

Nine months is a clinically important difference in the time for HIV-infected patients to delay initiation of lifelong HAART. During this time, patients could avoid the costs, toxicity risks, and resistance risks of ARVs. Because typical HAART regimens can cost $1200 per month compared with only $150 per month for valacyclovir, this strategy could result in an annual net savings of nearly $10000 per patient based on medication costs alone. Further, although the toxicity profile of current ARVs is significantly better than in earlier years of the HIV epidemic, long-term toxicities remain unknown because most of the agents in current use were approved within the last 5-10 years. The ARV resistance risks to be avoided are also considerable; in clinical trials evaluating efavirenz-based HAART regimens, the current 'gold standard' for first-line ARV therapy, 30-70% of the 15-20% patients experiencing virologic failure at one year have evidence of resistance to the NNRTI class of drugs, effectively eliminating the potential for using standard agents in this class thereafter[73-77]. Delaying HAART also provides time for patients to prepare psychologically for starting HAART, which is an important predictor of subsequent adherence[78,79]. Nine months is also the time between 3 quarterly appointments, allowing ample time for clinician and patient to prepare for HAART. Finally, although there is theoretical risk of developing drug-resistant HSV with chronic valacyclovir, this risk is minimal even in immunocompromised patients. Resistance rates have stayed stable at 5-6% despite increasing population exposure to related drugs over 2 decades, perhaps because it imposes a fitness cost on the virus[15,80-83]. Resistant HSV is occasionally seen in advanced HIV patients with persistent lesions despite therapy, not in asymptomatic shedding. For safety, those with frequent recurrences unresponsive to therapy during the trial will have their viral isolates tested for resistance.

### Interim Analysis

A single interim efficacy analysis is planned when 50% of the study endpoints have occurred; or at 3.5 years of follow-up, whichever occurs first. The DSMC will be asked to advise on early discontinuation of the trial at this point if there is clear evidence of benefit or harm in the treatment arm as defined below, using a two-sided statistical significance threshold of 0.0054 and power of 80%[70]. This analysis will have sufficient power to determine whether the intervention arm can expect a prolongation of the primary outcome from 2 years to 3.42 years. Based on the modest impact of HSV-2 and valacyclovir on HIV viral load, it is unlikely that such clear evidence of benefit will be seen at this interim analysis.

### Primary Analysis

The primary outcome of the trial is the time from baseline until reaching the primary endpoint, a composite of either a CD4 cell count ≤ 350 cells/mm^3 ^measured on two consecutive occasions at least 1 month apart, or initiation of HAART for any reason, whichever occurs first. The primary analysis will be a proportional hazards model, stratified by site, which will estimate the relative risk of progression to this endpoint associated with valacyclovir. Participants who do not reach either endpoint will be censored at the date of last CD4 count. The primary analysis will be conducted at the conclusion of the trial using the α = 0.0492 level of significance. This significance level adjusts for one interim analysis of the data according to the method of O'Brien and Fleming[70].

### Secondary Analyses

Random effects models will be used to compare the annual rate of change in the absolute CD4 cell count, the annual rate of change in the CD4 cell count percentage, and the median log_10 _HIV viral load between study arms. The rate of drug-related adverse events per year of follow-up, the number of episodes of HSV reactivations per year occurring at any anatomic site, the proportion of microbiologically confirmed flares of HSV during the trial that are caused by laboratory-confirmed acyclovir-resistant HSV, and the median overall quality of life score [84] at each 6-monthly time point will be compared between groups using Wilcoxon rank sum tests. All analyses will be by intention to treat, meaning that all participants will be analysed in the groups to which they were randomized.

Efforts to ensure that the CD4 and other trial data are well collected include biannual investigator meetings to review study procedures, regular site monitoring visits, and electronic data checks at the CTN National Data Centre. Because the analyses on rates of change in CD4 will only include CD4 counts prior to HAART initiation, the usual problem in HIV clinical trials of missing data among patients doing poorly clinically and dropping out of the trial is not anticipated to be an issue. Patients doing poorly clinically will reach the primary outcome, and it is only their CD4 counts before this time that will be included in the analysis.

### Subgroup Analyses

Three sets of subgroup analyses of the primary outcome will be performed. First, analysis will be conducted according to serological evidence of dual HSV-2 and HSV-1 infection, compared with only HSV-2 infection. These exploratory data will contribute to the emerging literature on the prognostic significance of HSV-1 in HIV co-infection[85]. Second, subgroup analyses will also be performed to determine whether participants with higher levels of adherence achieve greater benefits than those with lower adherence, recognizing that such associations may be partially driven by unmeasured confounders such as health-seeking behaviours. Third, subgroup analyses will also be performed according to country (Canada vs Brazil vs Argentina) since small differences related to clinical practice patterns, gender mix, clade or unknown factors may exist.

## Discussion

Given that HSV-2 is known to increase HIV viral load, that biological mechanisms of this interaction have been identified, and that pharmacologic suppression of HSV-2 has been shown to cause reciprocal decreases in HIV viral load, the hypothesis underlying the VALIDATE trial is that valacyclovir 500 mg twice daily may attenuate HIV disease progression and delay the need for initiating HAART in HIV-1, HSV-2 co-infected adults. Some data further suggest that acyclovir and, by extension, related drugs like valacyclovir, may have direct anti-HIV activity[86,87]. Some investigators have already advocated for all HIV patients to be routinely screened for HSV-2, with all co-infected individuals being offered chronic suppressive therapy in the hopes of attenuating rates of CD4 count decline[15]. Because acyclovir and its pro-drugs are safe, well-tolerated, and widely available, their use in this setting is realistic if benefit can be shown. However, type-specific HSV serologic testing is not yet routinely available, and anti-HSV drugs add to pill burden and cost, so demonstration of their effectiveness in randomized, controlled clinical trials is necessary before recommending widespread use. Although the Partners in Prevention trial recently demonstrated that HSV-2 suppression with acyclovir 400 mg twice daily results in a modest decrease in HIV disease progression in a cohort of co-infected adults in Sub-Saharan Africa,[45] the VALIDATE trial will examine this question in industrialized and middle-income countries where HIV clade, comorbid conditions, and standards of HIV care differ. Further, the intervention in the current trial is valacyclovir, the pro-drug of acyclovir, which has improved bioavailability relative to acyclovir[52].

It is recognized that HIV treatment guidelines are continuously under evaluation. In the time since the VALIDATE trial was designed, some panels have recommended that HAART should be initiated earlier, at the CD4 cell count threshold of 500 cells/mm^3 ^instead of 350 cells/mm^3^[6,9]. However, other guideline panels reviewing the same data have not reached the same conclusion,[7,8] suggesting there remains clinical equipoise regarding the optimal timing for initiating therapy.

This ongoing debate should not threaten the scientific integrity of the VALIDATE study for several reasons. First, the primary endpoint of this study was intentionally designed as a composite of not only meeting the CD4 threshold of 350 cells/mm^3^, but also the initiation of HAART for any reason, including patient or provider preference for earlier treatment, whichever occurs first. Second, randomization in this trial will be stratified by site to ensure that variation in practice regarding initiation of HAART across sites is distributed equally between treatment arms. Importantly, because no definitive clinical trial addressing the question of when to start therapy at thresholds of 350 cells/mm^3 ^or higher is anticipated within the next several years, the primary objective of the VALIDATE trial remains highly clinically relevant. Even in an era when therapy has been broadly recommended at a CD4 threshold of 350 cells/mm^3^, the median CD4 count at treatment initiation in industrialized countries has been 200-300 because of costs, patient preference, and concerns about toxicities, adherence and resistance. If shown to be beneficial, HSV-2 suppression could thus offer important salutary effects to individuals deemed eligible for HAART in the interim period while arrangements are made for medication coverage and while patients are counseled about the risks and benefits of antiretroviral therapy.

A related concern is that delays in the time until initiation of HAART could theoretically result in greater population-level risks of onward HIV transmission owing to increased population viral load. If demonstrated to be effective, use of the VALIDATE intervention will thus require careful consideration of its broader implications, and physicians will need to balance the personal clinical priorities of their patients with those of society at large. However, implementation of the VALIDATE intervention would not preclude bolstering of other interventions to curb the spread of HIV infection. Further mitigating this concern is the hypothesis that HSV-2 suppression, if implemented using an appropriate regimen and/or in appropriate populations, could have meaningful effects on decreasing HIV transmission, even if existing acyclovir-based clinical trials have been unsuccessful thus far[88,89].

Valacyclovir for HSV-2 suppressive therapy is an attractive potential intervention for patients with HIV-1, HSV-2 co-infection at early stages of HIV disease, and the VALIDATE trial is hypothesized to validate this potential for clinical benefit.

## Competing interests

The authors declare that they have no competing interests.

## Authors' contributions

DHST and SLW conceived the original idea for the trial. DHST, SLW, RK, JMR, BG and PC contributed to the design of the trial. JMR and DHST performed the sample size calculations for the trial. DHST wrote the original version of the manuscript, and all authors critically reviewed and approved the final version.
